# An observational study of asymmetry in CMT1A

**DOI:** 10.1136/jnnp-2014-309096

**Published:** 2014-10-13

**Authors:** Ana L Pelayo-Negro, Aisling S Carr, Matilde Laura, Mariola Skorupinska, Mary M Reilly

**Affiliations:** 1 MRC Centre for Neuromuscular Diseases, UCL Institute of Neurology, London, UK; 2 Service of Neurology, University Hospital “Marqués de Valdecilla (IDIVAL)”, University of Cantabria (UC) and “Centro de Investigación Biomédica en Red de Enfermedades Neurodegenerativas (CIBERNED)”, Santander, Spain

**Keywords:** NEUROPATHY, CLINICAL NEUROLOGY, NEUROGENETICS

Charcot-Marie-Tooth (CMT) disease is a clinically and genetically heterogeneous group of inherited neuropathies that was first described in 1886. CMT1A is the commonest form of CMT and accounts for 70% of demyelinating CMT (CMT1).^1^ It is an autosomal dominant neuropathy due to a 1.4 Mb duplication or rarely a triplication on chromosome 17p11.2 that contains the peripheral myelin protein 22 (PMP22) gene. Potential pathogenic mechanisms include alterations in protein homoeostasis, cholesterol dysregulation, increased calcium influx via P2X7 upregulation resulting in dysmyelination and/or disruption of axon-glia interactions contributing to axonal loss.^2^ The characteristic phenotype delineated by Harding and Thomas is of childhood-onset distal wasting, weakness and sensory loss, which progresses slowly over decades in a length-dependent, symmetrical manner.^3^ One study of paediatric CMT reported asymmetry of foot alignment and ankle flexibility in 3–5% of patients and found that asymmetry was associated with greater overall neuropathy severity according to CMTNS scale but there was no breakdown according to specific CMT genotypes.^4^ Careful clinical descriptions from many large CMT1A cohorts have not reported significant asymmetry in muscle strength or sensory deficits.^5^ ^6^

However, rare cases of superimposed inflammatory neuropathy or radiculopathy, either secondary to degenerative spinal disease or as a complication of enlarged nerve roots, have been reported in CMT1A. There has been one reported case of marked asymmetry with unilateral footdrop without an alternative explanation despite extensive assessment in a 19-year-old male patient.^7^ Interestingly, this patient was one of a pair of monozygotic twins with CMT1A who had similar electrophysiological markers of the disease but significant differences in semeiology. In practice, it is important to know how much asymmetry is allowed in this condition so as not to assume that asymmetry always suggests a differential or coincidental diagnosis. No study to date has systematically examined this issue.

We performed a retrospective case series review in a cohort of patients with clinically and genetically confirmed CMT1A followed up at least annually at the inherited neuropathy clinic in the National Hospital for Neurology and Neurosurgery. Routine practice involves annual clinical assessment using the CMT Examination Score (CMTES), which includes Medical Research Council (MRC) scoring of muscle strength and validated scales for quantification of pinprick (PP) and vibration sensation (VS) deficit .We recorded presence of asymmetry in strength in the commonly affected *first dorsal interosseous* (FDIO) and *abductor pollicis brevis* (APB) muscles in the upper limbs (UL) and ankle dorsiflexion and plantarflexion in the lower limbs (LL). Asymmetry was defined as a difference of greater than 1 MRC grade in contralateral muscles (without differentiation between grade 4−, 4 and 4+) and asymmetry of sensation as a difference of 1 point on the CMTES PP and vibration scales. We excluded patients with electrophysiological evidence of superimposed compressive neuropathy and those with examination findings suggesting significant foot, ankle and hip problems or spinal deformities, which are commonly associated orthopaedic complications in inherited neuropathy that can contribute to non-neuropathic motor deficits.

One hundred and eighty patients were included in this review, 40.1% male, mean (SD) age at examination was 41.7 (14.8) years. Asymmetry of strength was seen in 38 patients (21.1%), of whom 24 (13.3%) had asymmetrical UL and 17 (9.4%) asymmetrical LL strength. The distribution of asymmetry in UL was as follows: FDIO (range: 1–2 points on MRC scale) in 13 patients (7.2%) and APB (range: 1–3 points) in 14 patients (7.8%). In the LL: ankle dorsiflexion (range: 1–2 points) in 17 (9.4%) and ankle plantarflexion (range: 2–3 points) in two patients (1.1%; table 1). In three patients asymmetry was noted in both upper and LL; weaker left FDIO and right ankle dorsiflexion in one patient, weaker right APB and right dorsiflexion in a second, and right FDIO and right ankle dorsiflexion in the third. Patients with motor asymmetry were older (mean (SD)=49.2 (12.9) years with asymmetry; 39.35 (14.48) years without asymmetry; (p<0.005) and had a more severe neuropathy (mean (S.D.) CMTES=13.4 (4.7) with asymmetry, 9.7 (4.5) without asymmetry; (p=0.000). There was no correlation between weaker side and handedness (r=0.11, p=0.52). The proportion of affected relative to probands were similar in both groups; 32.3% probands in symmetrical and 36.8% in asymmetrical patients (p=0.49).

**Table 1:** Number of patients with motor and/or sensory asymmetry according to differences in MRC and CMTES sensory scoring systems

Difference	1	2	3	4
MRC score				
FDIO	10	3	1	0
APB	10	3	0	0
ADF	11	6	0	0
APF	0	1	1	0
CMTES sensory score				
PP	5	1	1	0
VS	0	2	6	1

ADF, ankle dorsiflexion; APB, abductor pollicis brevis; APF, ankle plantar flexion; CMTES, Charcot-Marie-Tooth Examination Score; FDIO, first dorsal interosseous; MRC, Medical Research Council; PP, pinprick; VS, vibration sense.

In terms of sensation, asymmetry was noted in 23 patients (12.8%); PP asymmetry (range: 1–3 points on CMTES scale) in nine patients (5%) and VS (range 2–4) in 10 (5.6%; table 1). Asymmetry in sensory deficit was more common in the LL 20/23 than in the UL (3/23). In patients with asymmetry, alternative pathologies were ruled out through careful clinical review by an experienced consultant neurologist (MMR), and directed electrophysiology and neuroimaging (MRI of the brain, spinal cord, nerve roots±plexii). All differences reported were attributed to CMT1A. The evolution of these signs and paraclinical evidence suggests this finding as compatible with the natural history of the condition, a phenomenon which has not been previously reported. It may be that dynometry and/or routine electrophysiological examination of all four limbs may increase sensitivity for subtle asymmetry in CMT1A but was beyond the scope of this observational clinical study.

In conclusion, we observed motor and sensory asymmetry in a significant minority of patients in this well phenotyped CMT1A cohort. The presence of asymmetry alone in CMT1A does not necessarily suggest that an alternative explanation needs to be sought.

## References

[1] Murphy SM Laura M Fawcett K Charcot- Marie-Tooth disease: frequency of genetic subtypes and guidelines for genetic testing. J Neurol Neurosurg Psychiatry 2012;83:706–10. 22577229 10.1136/jnnp-2012-302451 PMC3736805

[2] Li J Parker B Martyn C The PMP22 gene and its related diseases. Mol Neurobiol 2013;47:673–98. 23224996 10.1007/s12035-012-8370-x PMC3594637

[3] Harding AE Thomas PK The clinical features of hereditary motor and sensory neuropathy types I and II. Brain 1980;103:259–80. 7397478 10.1093/brain/103.2.259

[4] Burns J Ouvrier R Estilow OT Symmetry of foot alignment and ankle flexibility in paediatric Charcot-Marie-Tooth disease. Clin Biomech (Bristol, Avon) 2012;27:744–7. 10.1016/j.clinbiomech.2012.02.006 PMC3389135 22424781

[5] Birouk N Gouider R Le Guern E Charcot-Marie-Tooth disease type 1A with 17p11.2 duplication. Clinical and electrophysiological phenotype study and factors influencing disease severity in 119 cases. Brain 1997;120:813–23. 9183252 10.1093/brain/120.5.813

[6] Marques W Jr Freitas MR Nascimento OJ 17p duplicated Charcot-Marie-Tooth 1A. Characteristics of a new population. J Neurol 2005;252:972–9. 15765265 10.1007/s00415-005-0797-9

[7] García CA Malamut RE England JD Clinical variability in two pairs of identical twins with the Charcot-Marie-Tooth disease type 1A duplication. Neurology 1995;45:2090–3. 7501164 10.1212/wnl.45.11.2090

